# Untargeted Metabolomics‐Based Analysis of Metabolic Differences in Different Parts of *Bidens bipinnata* L.

**DOI:** 10.1002/cbdv.71819

**Published:** 2026-10-15

**Authors:** Shuya Xu, Jiaxin Dong, Fayin Tang, Lihong Li, Yunze Guo, Lijie Zuo

**Affiliations:** ^1^ College of Veterinary Medicine Henan University of Animal Husbandry and Economy Zhengzhou China

**Keywords:** *Bidens bipinnata* L., tissue‐specific metabolic differences, untargeted metabolomics, UPLC‐MS

## Abstract

*Bidens bipinnata* L. is a medicinal plant widely used in traditional Chinese medicine, yet the metabolic differences among its aerial parts remain largely unexplored. In this study, untargeted metabolomics based on ultra‑performance liquid chromatography‑‐mass spectrometry (UPLC‑‐MS) was employed to comprehensively compare the metabolite profiles of stems, leaves, and fruits, with six biological replicates per tissue. A total of 1028 metabolites were putatively annotated. Multivariate analysis, including principal component analysis (PCA) and orthogonal partial least‑squares discriminant analysis (OPLS‑DA), revealed distinct tissue‐specific metabolic profiles. Flavonoids such as luteolin, kaempferol‐3‐O‐rutinoside, and quercetin were significantly enriched in leaves. Differential metabolites were primarily enriched in flavone and flavonol biosynthesis, phenylpropanoid biosynthesis, and aromatic amino acid metabolism pathways, with distinct enrichment patterns across the three pairwise comparisons. These findings elucidate the tissue‐specific accumulation patterns of secondary metabolites in *B. bipinnata*, providing a theoretical basis for the targeted utilization of different plant parts and supporting its potential development as a functional feed additive in animal husbandry.

## Graphical abstract

Untargeted UPLC‐Q‐TOF‐MS metabolomics reveals that stems, leaves, and fruits of *Bidens bipinnata* L. harbor strikingly distinct metabolic landscapes. Among 1028 annotated metabolites, flavonoids including luteolin, quercetin, and kaempferol‐3‐O‐rutinoside are preferentially enriched in leaves via flavone and flavonol biosynthesis pathways. These tissue‐specific findings provide a metabolic blueprint for deploying this herb as a functional feed additive in animal husbandry.

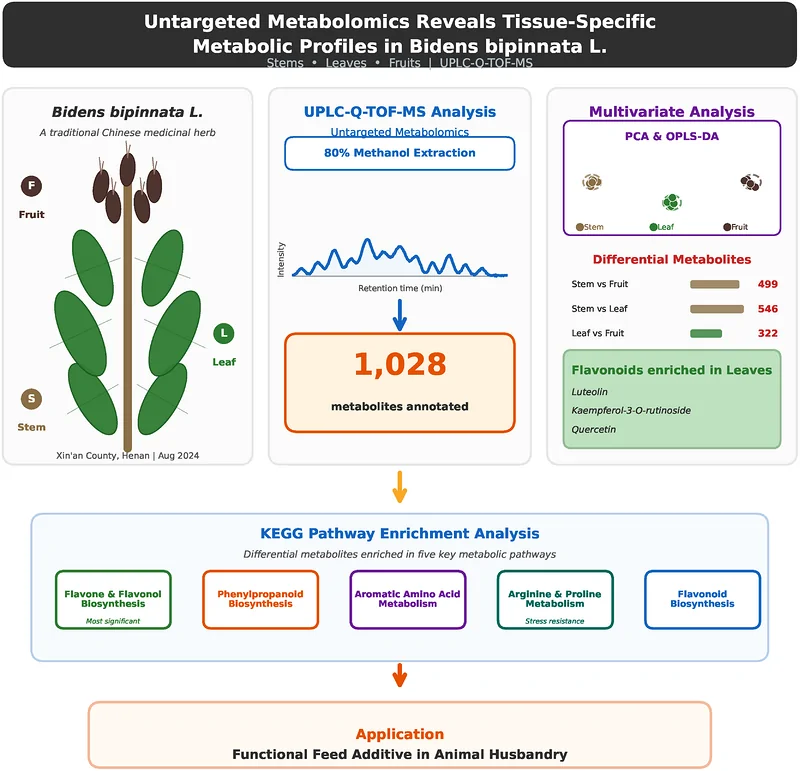

## 1 Introduction

*Bidens bipinnata* L., an annual herbaceous plant belonging to the genus *Bidens* within the Asteraceae family, is widely distributed across tropical and temperate regions worldwide [1, 2]. It has a long history of traditional medicinal use in China. According to the principles of traditional Chinese medicine, it possesses properties that clear heat, detoxify the body, alleviate diarrhea, and promote blood circulation to disperse blood stasis [3, 4]. Modern phytochemical studies have revealed that *B. bipinnata* L. contains a variety of bioactive compounds, including flavonoids, phenolic acids, polyacetylenes, and volatile oils, exhibiting a range of pharmacological activities such as anti‐inflammatory, antibacterial, and antioxidant effects [5]. Furthermore, the plant is nutritionally rich, characterized by high contents of proteins, trace elements, and vitamins, indicating its potential for development as a functional feed resource [6]. Preliminary investigations into its application in livestock and poultry farming have demonstrated promising prospects for its use in preventing gastrointestinal diseases and enhancing immune function.

However, both traditional clinical applications and existing research have predominantly utilized the whole plant of *B. bipinnata* L., leaving the differences in chemical composition among different plant parts (stems, leaves, and fruits) largely unclear. Due to variations in tissue function and physiological metabolic requirements, the biosynthesis and accumulation of secondary metabolites often differ significantly across plant parts [7]. Metabolomics enables comprehensive, high‐throughput qualitative and quantitative analysis of small‐molecule metabolites and has been widely employed to investigate differences in phytochemical composition and to discover bioactive components [8]. A widely used untargeted metabolomics approach based on ultra‐performance liquid chromatography‐mass spectrometry (UPLC‐MS) offers advantages such as high sensitivity and broad metabolite coverage, making it particularly suitable for elucidating differences in metabolite profiles and accumulation patterns across distinct tissues [9, 10]. In this study, untargeted metabolomics was employed to analyze differential metabolites and characterize the associated metabolic pathways in the stems, leaves, and fruits of *B. bipinnata* L. The findings aim to enhance the resource utilization efficiency of this plant and provide a theoretical foundation for its development as a functional feed additive, as well as for the efficient utilization of its resources.

## 2 Results and Discussion

### 2.1 Total Ion Current Chromatogram Analysis

The base peak intensity (BPI) chromatograms of the stem, leaf, and fruit parts of *B. bipinnata* L. in positive ion mode are shown in Figure S1. The chromatographic peaks of each sample were well‐resolved, with stable baselines and evenly distributed peaks, indicating that the instrument operated stably with minimal systematic error, thereby yielding reliable data. During metabolite detection, the mass spectrometry signal intensity remained stable with good reproducibility, ensuring the quality of data acquisition and providing a reliable foundation for subsequent differential metabolite screening and quantitative analysis.

### 2.2 Differential Metabolites Analysis

#### 2.2.1 Multivariate Statistical Analysis

Principal component analysis (PCA), an unsupervised pattern recognition method, was employed to reveal the internal structure of multiple variables through several principal components and to reflect the original state of the metabolomic data, allowing preliminary assessment of metabolic differences among sample groups and intra‐group variation [11]. PCA was performed on metabolites from different parts of *B. bipinnata* L., and the results are shown in Figure 1. The interpretability rate of the model, R^2^X, was 0.603 (>0.5), with the variance contributions of PC1 and PC2 being 36.9% and 28.6%, respectively, indicating good model fitting and stability. Samples from different parts of *B. bipinnata* L. exhibited clear separation, suggesting significant differences in metabolite composition among the stem, leaf, and fruit (Figure 1A).

**FIGURE 1:**
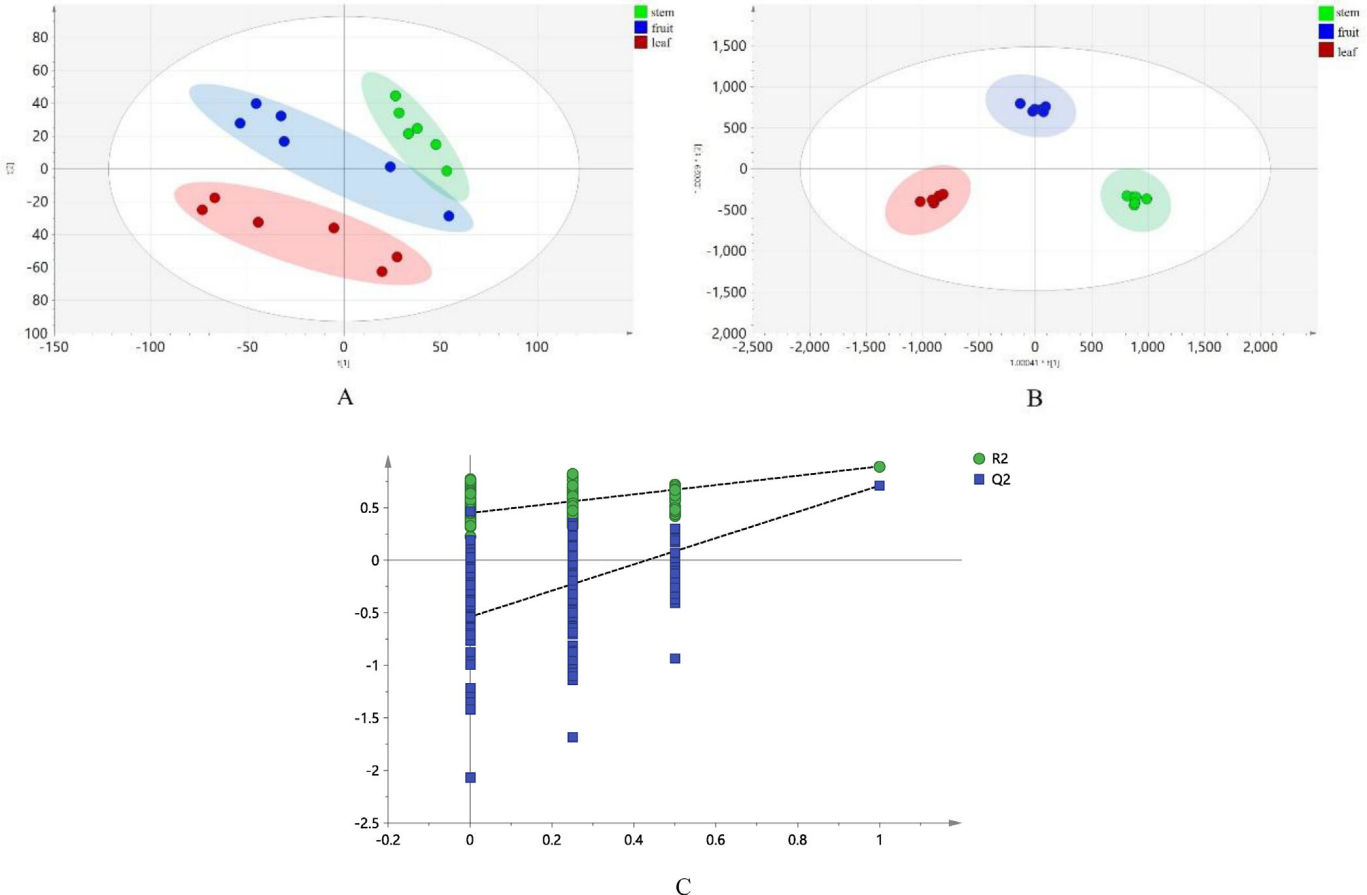
Multivariate statistical analysis of metabolites from different parts of *B. bipinnata* L. (A) PCA score plot. (B) OPLS‐DA score plot. (C) Permutation test.

Orthogonal partial least squares discriminant analysis (OPLS‐DA), a supervised multivariate statistical method, was further conducted to address the insensitivity of PCA to variables with low correlation and to effectively eliminate irrelevant factors, thereby highlighting intergroup differences and facilitating differential metabolite screening [12]. The OPLS‐DA results demonstrated strong clustering within each group and clear separation among different groups (Figure 1B), further confirming significant metabolic differences across the three parts. The model parameters (R^2^X = 0.883), (R^2^Y = 0.993), (*Q* ^2^ = 0.907) indicated robust predictive ability and model reliability, supporting its applicability for analyzing metabolites from different parts of *B. bipinnata* L. Furthermore, to validate the OPLS‐DA model and guard against overfitting risk, a permutation test with 200 iterations was performed. The regression lines yielded intercepts of *R* ^2^ = 0.449 and *Q* ^2^ = −0.54, demonstrating that the established OPLS‐DA model was robust, valid, and free from overfitting, thereby ensuring the reliability of differential metabolite screening.

#### 2.2.2 Analysis of Differential Metabolite Changes

A total of 1028 metabolites were putatively annotated from different parts of *B. bipinnata* L. Metabolite annotations were evaluated according to metabolomics standards initiative (MSI) criteria; without authentic standards, no Level 1 assignments were made, whereas robust tandem mass spectrometry (MS/MS) matches (≥70) and mass‐only inferences were classified as Levels 2 and 3, respectively. Differential expression analysis of all metabolites was performed based on multivariate statistical analysis. Differential metabolites satisfying variable importance in projection (VIP) > 1, *p* < 0.05, false discovery rate (FDR) *q* < 0.05, and |log2FC| ≥ 1 were visualized using volcano plots (see Table S1 for detailed statistics). As shown in Figure 2, 316 up‐regulated and 183 down‐regulated metabolites were putatively annotated in the stem versus fruit group, resulting in 499 differential metabolites; in the stem versus leaf group, 300 up‐regulated and 246 down‐regulated metabolites were putatively annotated, totaling 546 differential metabolites; in the leaf versus fruit group, 184 up‐regulated and 138 down‐regulated metabolites were putatively annotated, totaling 322 differential metabolites. These differences and variations in metabolites are of great significance for elucidating the applications of different parts of *B. bipinnata* L. Representative differential metabolites are listed in Table 1.

**FIGURE 2:**
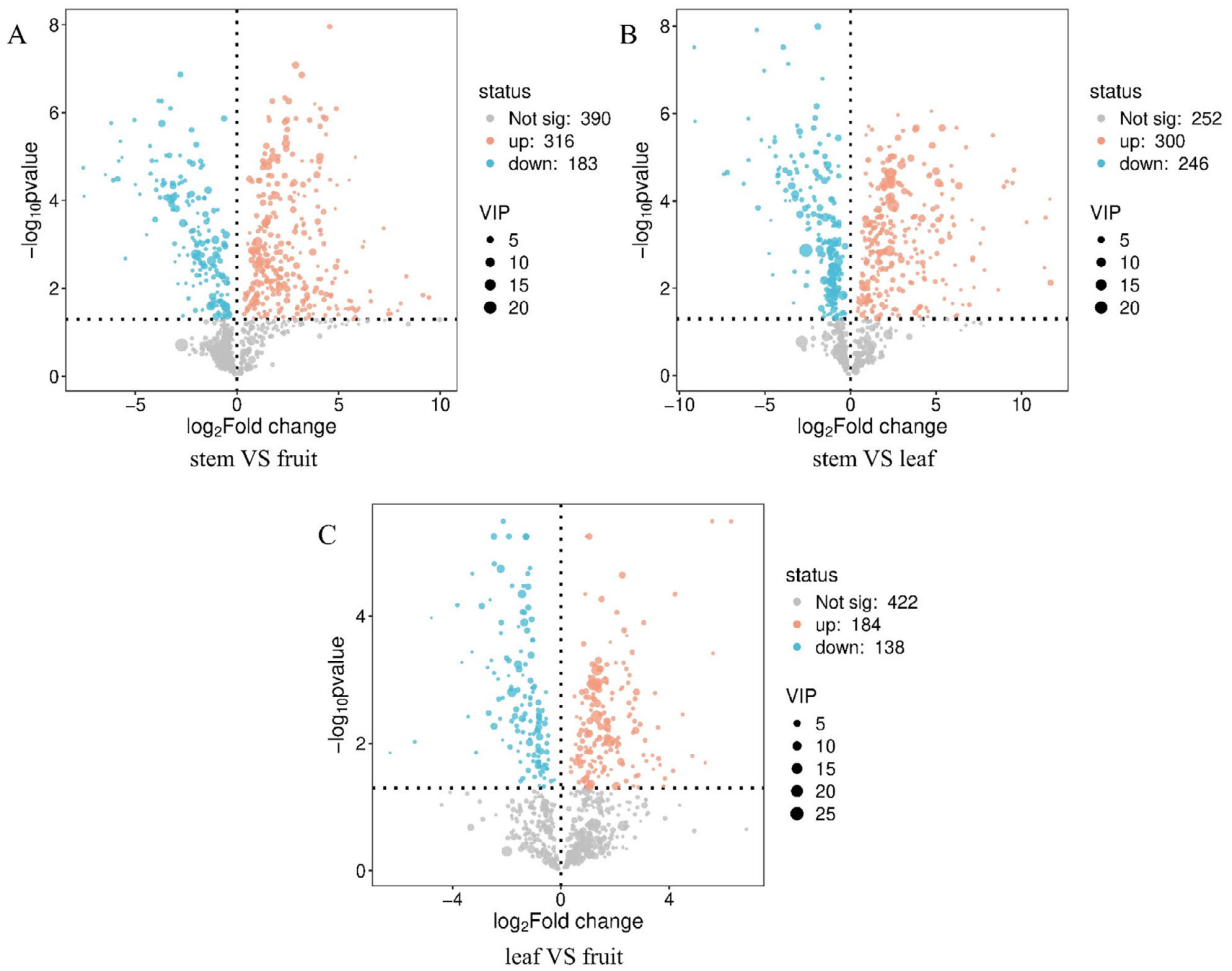
Volcano plots of differential metabolites among different parts of *B. bipinnata* L. (A) Stem versus fruit. (B) Stem versus leaf. (C) Leaf versus fruit.

**TABLE 1:** Summary of representative differential metabolites.

No.	Component name	Observed *m/z*	Observed retention time (RT, min)	Mass error (mDa)	Formula	Adducts	MS/MS fragment ions
1	L‐Proline	116.0707	0.64	−0.45	C_5_H_9_NO_2_	+H	100.0756,70.0657
2	L‐Arginine	175.1182	0.76	−1.30	C_6_H_14_N_4_O_2_	+H	158.0912,130.0963,116.0807,70.0651,60.0552
3	L‐Tyrosine	182.081	0.92	−0.72	C_9_H_11_NO_3_	+H	136.0762,119.0494,147.0438
4	Protocatechuic acid	155.0337	0.95	−0.73	C_7_H_6_O_4_	+H	137.0233,127.0389,109.0287
5	Isoleucine	132.1023	1.13	−0.15	C_6_H_13_NO_2_	+H	86.0961,69.0694,57.0699
6	L‐Phenylalanine	166.086	1.56	−0.80	C_9_H_11_NO_2_	+H	120.0808,103.0543,91.0548,77.0391
7	L‐Tryptophan	205.09727	1.78	−0.43	C_11_H_12_N_2_O_2_	+H	188.0755,159.0952,118.0645,130.0645
8	Vanillic acid	169.0496	2.76	−0.48	C_8_H_8_O_4_	+H	151.0389,125.0597,107.0491
9	(6E,12E)‐tetradecadiene‐8,10‐diyne‐1,3,14‐triol	273.0877	2.89	−1.60	C_14_H_18_O_3_	+K	235.1194,217.1088,181.0876
10	Quercetagetin	319.0437	3.98	−1.69	C_15_H_10_O_8_	+H	301.0335,273.0386,153.0178,121.0281
11	*β*‐Sitosterol	453.3496	5.64	−0.27	C_29_H_50_O	+K	414.3862,369.3474,303.2317,69.0699
12	7‐Phenyl‐hepta‐4,6‐diyn‐1,2‐diol	199.0756	5.98	−0.30	C_13_H_10_O_2_	+H	181.0650,163.0544,135.0441,107.0491,77.0391
13	1,2‐Dihydroxytrideca‐3,5,7,9,11‐pentayne	197.0598	6.05	−0.45	C_13_H_8_O_2_	+H	179.0492,151.0542,123.0441,95.0491
14	Chalcone‐*α*,3,2',4'‐hydroxy‐2'‐O‐*β*‐D‐glucopyranoside	435.12933	6.1	0.21	C_21_H_22_O_10_	+H	273.0757,255.0651,153.0178,137.0233,121.0281
15	Kaempferol‐3‐O‐rutinoside	595.166	6.38	−0.30	C_27_H_30_O_15_	+H	287.0545, 259.0597,241.0491,177.0184,165.0184,133.0287,121.0281
16	Quercetin–dimethyl ether	331.0807	6.39	−1.08	C_17_H_14_O_7_	+H	316.0573,303.0499,285.0393,257.0444,153.0178
17	Astragalin	449.1064	6.69	−1.99	C_21_H_20_O_11_	+H	287.0545,269.0440, 213.0542,177.0184,165.0184,153.0178
18	7‐Phenyl‐2‐heptene‐4,6‐diyne‐1‐ol	185.0973	6.69	0.66	C_13_H_12_O	+H	167.0897,149.0601,135.0441,107.0491,77.0391
19	Centaurein	523.1442	6.7	−0.97	C_24_H_26_O_13_	+H	361.0914,287.0545,259.0597,153.0178
20	Naringenin	273.0787	6.89	2.40	C_15_H_12_O_5_	+H	255.0652,153.0178,147.0441,121.0281
21	Luteolin	287.0541	6.98	−1.46	C_15_H_10_O_6_	+H	269.0435,241.0486,213.0542,179.0340,153.0178,149.0597,133.0287
22	Luteoloside	449.1100	7.62	1.61	C_21_H_20_O_11_	+H	287.0545,241.0486,213.0542,153.0178,149.0597
23	Deca‐4,6,8‐triyne polyacetylene diglycoside	471.1886	7.62	1.96	C_22_H_30_O_11_	+H	387.06839,298.06908,145.05089,133.02905
24	Kaempferol	287.0545	7.63	−1.06	C_15_H_10_O_6_	+H	269.0440,231.0650,213.0542,177.0184,165.0184,133.0287,121.0281
25	Phenylheptatriyne (1‐phenylhepta‐1,3,5‐triyne)	187.0511	7.63	−1.27	C_13_H_8_	+Na	159.0441,133.0647,119.0491,91.0548,77.0391
26	tri‐O‐caffeoylquinic acid	701.1489	7.69	0.66	C_34_H_30_O_15_	+Na	539.1172,377.0914,341.0655,179.0340,161.0234
27	6‐Phenylhexa‐1,3,5‐triyn‐1‐yl acetate	209.0608	7.7	0.55	C_14_H_8_O_2_	+H	149.0601,121.0281,107.0491,77.0391
28	Ferulic acid	217.0472	7.74	−0.48	C_10_H_10_O_4_	+Na	163.03876,133.02868,135.04405
29	7‐Hydroxycoumarin	163.0389	7.75	−0.62	C_9_H_6_O_3_	+H	145.0283,117.0334,89.0385,69.0334
30	2‐*β*‐D‐Glucopyranosyloxy‐1‐hydroxyltrideca‐3,5,7,9,11‐pentryne	367.1744	7.75	−1.28	C_19_H_26_O_7_	+H	205.0500,187.0394,179.0492,161.0386,151.0542
31	Bidensynoside A1	381.1535	7.76	−1.44	C_19_H_24_O_8_	+H	219.1015,183.0804,163.0755,145.0505
32	2‐*β*‐D‐Glucopyranosyloxy‐1‐hydroxy‐trideca‐3,5,7,9,11‐pentayne	381.0936	7.84	−1.42	C_19_H_18_O_7_	+Na	219.042,201.031,163.076
33	Hesperidin	611.1976	7.87	0.00	C_28_H_34_O_15_	+H	303.0860,259.0755,177.0184,153.0178
34	Quercetin‐3‐O‐pentose	435.0941	8.88	1.36	C_20_H_18_O_11_	+H	303.0499,285.0393,257.0444,201.0546,121.0281
35	Quercetin 3‐O‐*β*‐D‐glucofuranoside	465.103	9.75	−0.30	C_21_H_20_O_12_	+H	303.0499,285.0393, 229.0495,201.0546,153.0178

*Note*: Isomeric compounds (e.g., astragalin vs. luteoloside; luteolin vs. kaempferol) were distinguished by combined evidence of distinct retention times and diagnostic MS/MS fragment ions. All annotations are at MSI Level 2 and require further confirmation with authentic standards.

To further visualize the changes in differential metabolites, the top 15 metabolites with the highest VIP values in each comparison group were subjected to visual analysis, with the results shown in Figure 3. Two‐way hierarchical clustering analysis was performed on the samples and differential metabolites, and the resulting heatmap is presented in Figure 4. The analysis results indicated pronounced differences in metabolite expression among different parts, with notable divergence in metabolite composition between the fruit and leaf groups.

**FIGURE 3:**
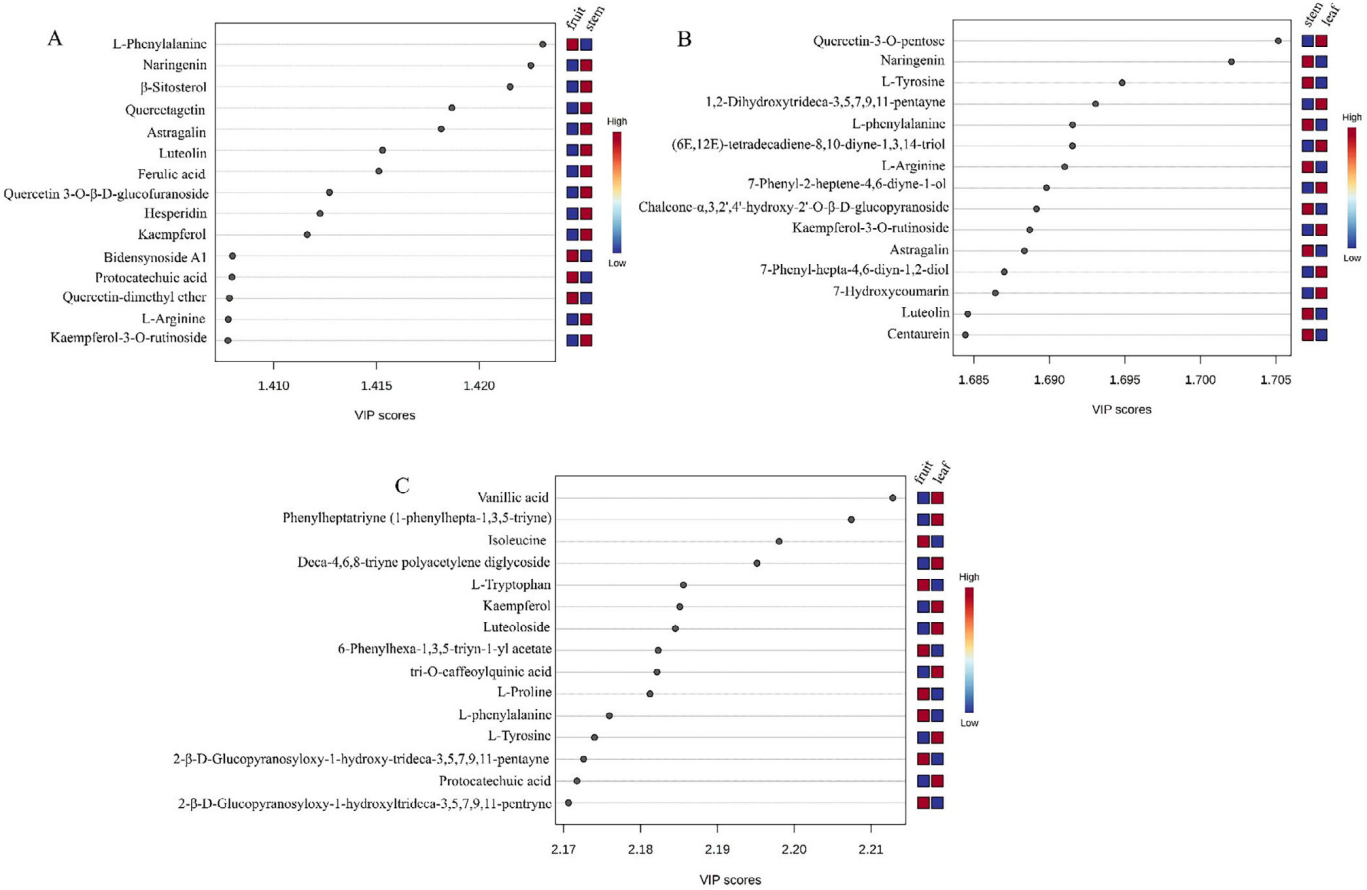
VIP scatter plot of the differential metabolites. (A) Fruit versus stem. (B) stem versus leaf. (C) fruit versus leaf.

**FIGURE 4:**
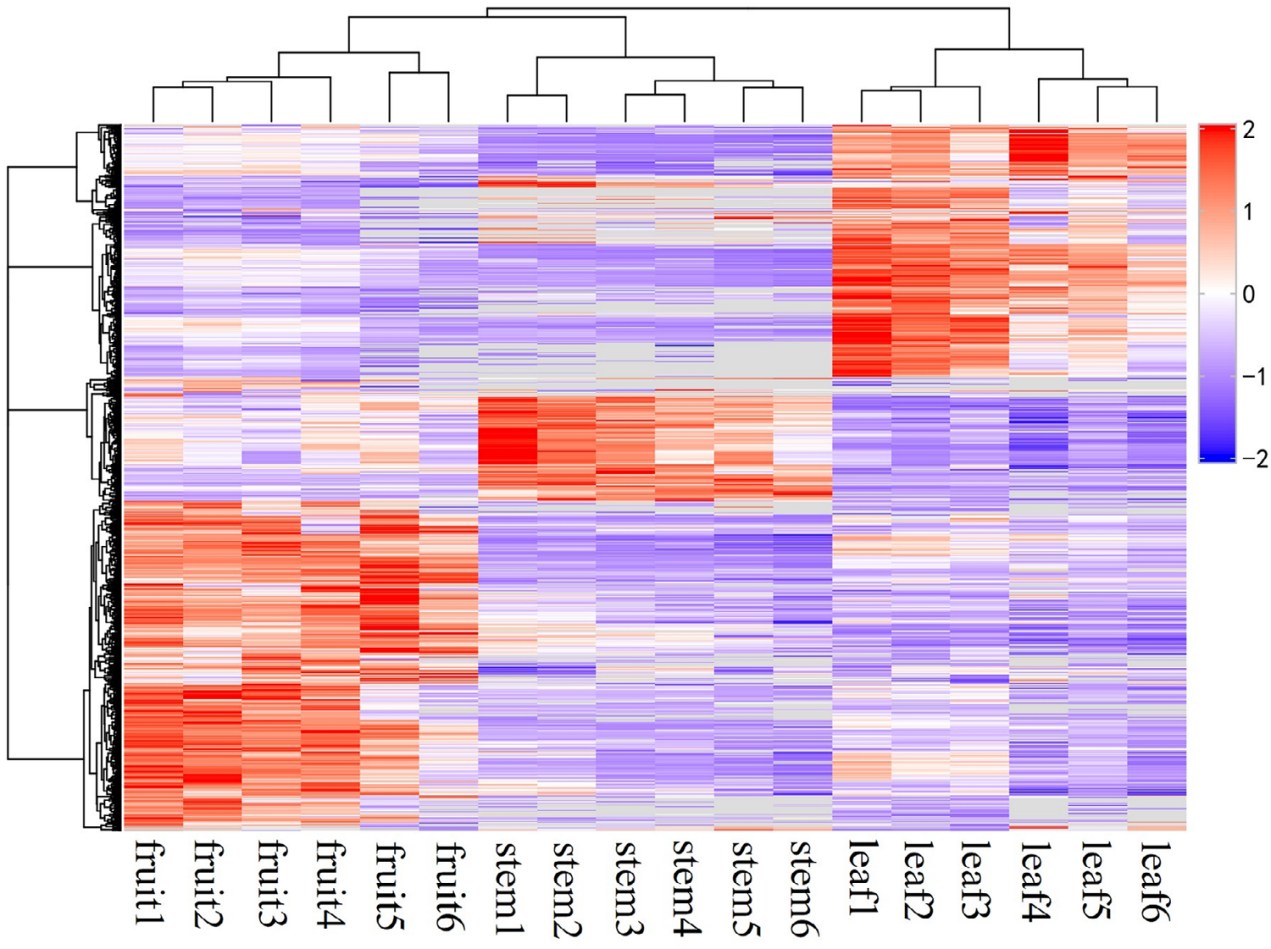
Correlation heatmap and cluster analysis of the differential metabolites.

#### 2.2.3 Metabolic Pathway Analysis of Differential Metabolites

Differential metabolites among different parts of *B. bipinnata* L. were annotated using the Kyoto Encyclopedia of Genes and Genomes (KEGG) database, followed by enrichment analysis of the annotated results. The top 10 metabolic pathways with the highest significance based on *p* value were selected for further analysis. As shown in Figure 5, in the stem versus fruit group, differential metabolites were primarily annotated and enriched in pathways such as flavone and flavonol biosynthesis, flavonoid biosynthesis, phenylpropanoid biosynthesis, tropane, piperidine and pyridine alkaloid biosynthesis, and phenylalanine metabolism (Figure 5A). In the stem versus leaf group, differential metabolites were mainly annotated in pathways including flavone and flavonol biosynthesis, phenylalanine, tyrosine and tryptophan biosynthesis, flavonoid biosynthesis, isoquinoline alkaloid biosynthesis, and tropane, piperidine and pyridine alkaloid biosynthesis (Figure 5B). In the leaf versus fruit group, differential metabolites were predominantly annotated in pathways such as phenylalanine, tyrosine and tryptophan biosynthesis, glucosinolate biosynthesis, indole alkaloid biosynthesis, isoquinoline alkaloid biosynthesis, tropane, piperidine and pyridine alkaloid biosynthesis, and phenylalanine metabolism (Figure 5C).

**FIGURE 5:**
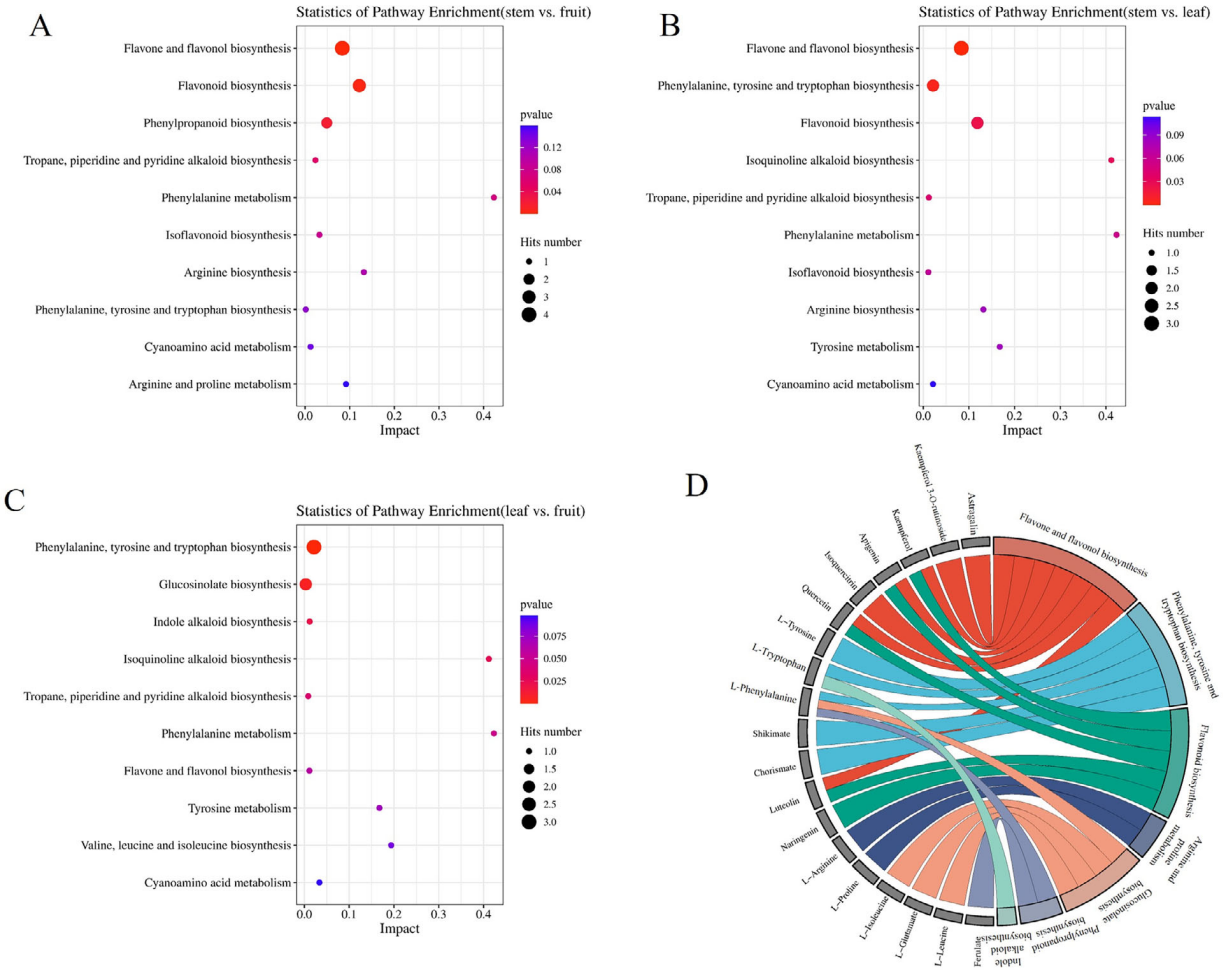
KEGG pathway enrichment bubble plots of differential metabolites between groups (A–C) and metabolite–pathway chord diagram among the three groups (D).

Pathway enrichment analysis was performed on the stem, leaf, and fruit groups, and the differential metabolites enriched in each pathway were visualized (Figure 5D). The results revealed that differential metabolites were significantly enriched in pathways including flavone and flavonol biosynthesis, phenylalanine, tyrosine and tryptophan biosynthesis, flavonoid biosynthesis, arginine and proline metabolism, glucosinolate biosynthesis, phenylpropanoid biosynthesis, and indole alkaloid biosynthesis.

## 3 Discussion

In this study, untargeted metabolomics based on UPLC‐Q‐TOF‐MS was employed to analyze the metabolites of different plant parts (stem, leaf, and fruit) of *B. bipinnata* L. A total of 1028 compounds were putatively annotated. PCA revealed a clear separation trend among samples from different parts, indicating significant differences in metabolite composition across the three parts. Further differential metabolite screening was performed using the OPLS‐DA model, and the results showed 499, 546, and 322 differential metabolites between stem and fruit, stem and leaf, and leaf and fruit groups, respectively. These findings suggest that the accumulation of chemical constituents in different parts of *B. bipinnata* L. exhibits pronounced tissue specificity, which is closely associated with the distinct physiological functions of each part.

### 3.1 Tissue Distribution Characteristics of Flavonoids

In this study, flavonoids were found to exhibit the most significant differences among different parts of *B. bipinnata* L., with both the variety and content of flavonoids being higher in the leaves. Compounds such as luteolin, kaempferol‐3‐O‐rutinoside, quercetin, and their glycoside derivatives were all present in higher abundance in the leaves. This distribution pattern is consistent with previous studies, which have indicated that the leaves of *Bidens* species are the primary sites for flavonoid synthesis and accumulation [13].

From the perspective of metabolic pathway enrichment, differential metabolites between leaves and fruits were significantly enriched in the flavone and flavonol biosynthesis and flavonoid biosynthesis pathways. This phenomenon can be explained from the perspective of biosynthetic mechanisms: as the primary organ for photosynthesis, the leaf is chronically exposed to environmental stresses such as strong light and ultraviolet radiation. According to previous literature, flavonoids possess the ability to absorb ultraviolet light and scavenge free radicals, serving as an important protective mechanism for plants against photo‐oxidative stress [14, 15]. This suggests that the high abundance of flavonoid metabolites in leaves observed in this study may reflect their physiological function and environmental adaptation.

Notably, glycosyltransferases play a key role in the tissue‐specific accumulation of flavonoids [16]. Previous studies have demonstrated that 11 candidate glycosyltransferase genes, including DN19630, are significantly more highly expressed in the leaves of *Bidens pilosa* L. than in the roots and stems. These glycosyltransferases are involved in the glycosylation modification of flavonoids, affecting their solubility and stability, thereby determining the distribution pattern of flavonoid metabolites across different tissues [17]. The enrichment of various flavonoid glycosides (such as kaempferol‐3‐O‐rutinoside and astragalin) in the leaves putatively annotated in this study is consistent with the expression patterns of the aforementioned glycosyltransferases.

### 3.2 Tissue Differences in Phenylpropanoid Metabolic Pathways

Enrichment analysis of differential metabolites revealed that the phenylpropanoid biosynthesis pathway was significantly enriched in both the stem versus fruit and leaf versus fruit comparisons. Phenylpropanoid metabolism represents a central pathway in plant secondary metabolism, yielding a wide range of bioactive constituents, including flavonoids, phenolic acids, and coumarins [18, 19]. In this study, phenolic acid components such as ferulic acid and protocatechuic acid exhibited significant differences among the different parts.

The initial substrate of the phenylpropanoid biosynthetic pathway is L‐phenylalanine, which is consistent with the detection of amino acid metabolites, including L‐phenylalanine and L‐tyrosine, among the differential metabolites putatively annotated in this study. From a metabolic network perspective, the phenylalanine, tyrosine, and tryptophan biosynthesis pathway was significantly enriched in all three comparison groups, suggesting that differences in upstream amino acid metabolism may directly drive the tissue‐specific accumulation of downstream secondary metabolites.

### 3.3 Potential Significance of Arginine and Proline Metabolism

In this study, the arginine and proline metabolism pathway was also found to exhibit significant differences among different parts of *B. bipinnata* L. This pathway is closely associated with plant stress resistance, with proline serving as an important osmolyte and antioxidant. Both L‐proline and L‐arginine were characterized as differential metabolites in Table 1, and their distinct distribution patterns between leaves and fruits may be related to the degree of oxidative stress encountered by different tissues [20, 21]. Previous studies have demonstrated that *Bidens* species possess notable antioxidant activity, and the arginine‐proline metabolism pathway may represent an important component of their antioxidant defense mechanism [22, 23, 24].

### 3.4 Association Between Metabolic Pathway Enrichment and Pharmacological Activities

The metabolic pathways enriched in this study, such as flavone and flavonol biosynthesis, phenylpropanoid biosynthesis, and indole alkaloid biosynthesis, are closely linked to the known pharmacological activities of *B. bipinnata* L. Flavonoids are the primary bioactive constituents responsible for the anti‐inflammatory, antibacterial, and antioxidant effects of *B. bipinnata* L.; phenolic acids derived from the phenylpropanoid pathway also exhibit potent antioxidant and anti‐inflammatory activities; indole alkaloids may contribute to its antibacterial and immunomodulatory effects [3, 25]. However, the pharmacological activities were not directly verified in the present study, and further experimental confirmation is warranted.

From the perspective of veterinary clinical application, the metabolite composition data obtained in this study may provide a preliminary basis for future investigations. First, the high abundance of flavonoids in the leaves suggests that the leaves of *B. bipinnata* L. may possess superior anti‐inflammatory and antioxidant activities, and might therefore be of potential relevance for the prevention and treatment of inflammatory diseases and oxidative stress‐related disorders in livestock and poultry. Second, the presence of polyacetylene compounds may confer unique antibacterial properties, with studies indicating that polyacetylene components from *Bidens* species exhibit inhibitory effects against various pathogenic bacteria in vitro [26]. Furthermore, previous research has demonstrated that seven marker components of *B. bipinnata* L. (gallic acid, protocatechuic acid, rutin, hyperoside, polyyne glycoside from *Bidens*, luteolin, and quercetin) are highly distributed in the liver [21], suggesting that *B. bipinnata* L. may exert hepatoprotective effects in livestock and poultry, thereby possessing application potential in the prevention and treatment of liver diseases.

### 3.5 Limitations and Future Perspectives

It should be acknowledged that the present untargeted metabolomics approach was designed for comparative profiling rather than definitive compound identification; all metabolite annotations were made without authentic standards, and thus require further confirmation via targeted analysis using reference compounds. Additionally, metabolite acquisition in this study was conducted exclusively in positive ion mode, which may have resulted in reduced detection sensitivity or even omission of certain acidic metabolites—such as organic acids and sulfated flavonoids—that typically exhibit higher ionization efficiency under negative ion mode. Future studies should therefore employ both positive and negative ion modes to achieve more comprehensive metabolite coverage. Nevertheless, the broad untargeted metabolomics platform utilized in this study is primarily optimized for the major active constituents of *B. bipinnata* L.—namely, flavonoid glycosides, alkaloids, and terpenoids—that demonstrate favorable ionization response in positive ion mode, thereby ensuring that the core scientific questions of this study can still be effectively addressed. Moreover, although the observed tissue‑specific accumulation patterns suggest potential pharmacological relevance, the actual bioactivities—such as anti‑inflammatory, antioxidant, and antimicrobial effects—were not experimentally verified in this study. Therefore, future investigations should include bioactivity‑guided fractionation, in vitro assays, and in vivo feeding trials in livestock to validate the efficacy, safety, and digestibility of *B. bipinnata* L. as a functional feed additive. Such studies will be essential to translate the current metabolomics findings into practical veterinary applications.

## 4 Conclusions

In this study, untargeted metabolomics was employed to systematically analyze the metabolic differences among the stems, leaves, and fruits of *B. bipinnata* L. The results demonstrated that the metabolite composition of different parts of *B. bipinnata* L. exhibits pronounced tissue specificity. Flavonoids (such as luteolin, kaempferol‐3‐O‐rutinoside, quercetin) were significantly enriched in the leaves. Differential metabolites were mainly enriched in pathways such as flavonoid biosynthesis, phenylpropanoid biosynthesis, aromatic amino acid metabolism, and arginine‐proline metabolism, revealing the patterns of secondary metabolite synthesis and accumulation across different tissues. This study provides a preliminary metabolite basis for the scientific application of *B. bipinnata* L. in animal husbandry and offers new insights for the comprehensive utilization of traditional Chinese medicinal resources.

## 5 Materials and Methods

### 5.1 Plant Materials

Specimens of *B. bipinnata* L. were collected in August 2024 from Xin'an County, Henan Province, China (latitude and longitude: 34°44′45.24″N, 112°3′19.48″E). A total of 18 healthy mature plants were sampled, with 6 individual plants collected for each of the three tissue types (stems, leaves, and fruits). At the time of collection, the plants were at the developmental stage spanning from full flowering to fruit ripening; fruits were harvested at full maturity, as indicated by visibly darkened pigmentation. The plant material was authenticated as *B. bipinnata* L. by Professor Li Lihong. The collected specimens were separated into stems, leaves, and fruits, and the fresh tissues were oven‑dried at 50°C for 48 h until constant weight was reached. The dried materials were ground into a fine powder and sealed in airtight containers until further extraction and analysis. The samples were stored in the Teaching and Research Office of Traditional Chinese Veterinary Medicine, College of Veterinary Medicine, Henan University of Animal Husbandry and Economy.

### 5.2 Reagents

Acetonitrile and formic acid (both of chromatographic grade) were purchased from Thermo Fisher Scientific (Waltham, MA, USA). Methanol (analytical grade) was obtained from Huacheng Industrial Development Co., Ltd. (Shanghai, China). Ultrapure water was prepared using a Milli‐Q water purification system (Billerica, MA, USA).

### 5.3 Sample Preparation and Extraction

An accurately weighed 0.5 g portion of sample powder was mixed with 10 mL of 80% methanol solution and subjected to ultrasonic extraction at room temperature for 40 min. The extract was filtered, and the filtrate was collected. The supernatant was then centrifuged at 12,000 rpm for 10 min at 4 °C, and the resulting supernatant was filtered through a 0.22 µm membrane filter prior to UPLC‐MS analysis.

### 5.4 UPLC–MS Conditions

Chromatographic experiments, including UPLC–MS analysis using a Waters Q‐TOF MS instrument, were performed as described previously [27, 28]. Chromatographic separation was conducted on a Waters BEH C18 column (100 × 2.1 mm, 1.8 µm) maintained at 40°C. The mobile phase consisted of acetonitrile (A) and 0.1% formic acid in water (B) with a gradient elution program as follows: 0–2 min, 6% A; 2–15 min, 6%–100% A; 15–15.1 min, 100%–6% A; 15.1–17 min, 6% A. The flow rate was set at 0.3 mL/min, and the injection volume was 5 µL.

Mass spectrometry was performed using an electrospray ionization (ESI) source in positive ion mode. The mass scan range was set to *m/z* 100–1000. The capillary voltage was 2.5 kV, the spray voltage was 2.8 kV, and the drying gas flow rate was 50 L/h. The collision energy was 15 eV, and the drying gas temperature was maintained at 350°C.

### 5.5 Quality Control

A series of quality control (QC) measures were implemented throughout the entire analytical sequence. A solvent blank (the extraction solvent injected without plant material) was used to monitor instrument background, and an extraction blank (carried through the full extraction procedure in the absence of sample) was used to detect potential contamination introduced during sample preparation. A pooled QC sample was prepared by mixing equal aliquots of all extracts and was injected after every 10 sample injections to evaluate instrument stability and data reproducibility. To avoid systematic bias, the injection order of all samples and QC samples was randomized. Prior to the formal analytical run, five conditioning injections of the pooled QC sample were performed to equilibrate the chromatographic column and ion source. The analytical stability and reproducibility were further assessed by PCA of all QC samples, and the results are presented in Figure S2.

### 5.6 Data Processing

The data acquired from Waters Q‐TOF MS were preprocessed using Waters MassLynx 4.1 software. The acquired spectra underwent peak extraction and alignment to generate a standardized data matrix comprising retention times and mass‐to‐charge ratios (*m/z*). The processed data were then imported into SIMCA‐P 13.0 (Umetrics, Sweden) for normalization and pattern recognition analysis, including PCA andOPLS‐DA. In addition, to assess the significance of differences among components from different parts of *B. bipinnata* L., a one‐way analysis of variance (ANOVA) was performed, with the significance level (*α*) set at 0.05. Differential metabolites among different parts were characterized based on the VIP derived from the OPLS‐DA model, fold change, *p* value, and FDR‐adjusted *q*‐value. To control the false discovery rate arising from multiple testing, raw *p* values were adjusted using the Benjamini–Hochberg (BH) method. A metabolite was retained as significantly differential only when it met all of the following criteria: VIP > 1, *p* < 0.05, FDR *q* < 0.05, and |log2 fold change (log2FC)| ≥ 1. The complete statistical parameters for the top 15 representative differential metabolites from each comparison (VIP, *p* value, FDR *q*‐value, and log2FC) are listed in Table S1. Pathway enrichment analysis was subsequently performed using KEGG.

### 5.7 Metabolite Annotation and Structural Identification

Metabolite annotation and structural identification were performed using a combination of in‑house and publicly available databases. The databases queried included the UNIFI database, MassBank (https://massbank.eu/MassBank/), HMDB (https://www.hmdb.ca/), and METLIN (https://metlin.scripps.edu/), along with an in‑house library of authentic reference standards. A match score threshold of ≥ 70 was applied for reliable annotation. Identification of metabolites was achieved by comparing retention time, molecular mass, MS/MS fragmentation spectra, and collision energy across different energy levels. In accordance with the MSI guidelines, all annotations were assigned to Level 2 (putatively annotated based on MS/MS spectral matching) or Level 3 (putatively characterized compound class based on accurate mass), as no authentic standards were employed for Level 1 confirmation. The differentially accumulated metabolites among stems, leaves, and fruits of *B. bipinnata* L. were further manually verified and subsequently categorized into stem, leaf, and fruit groups.

## Author Contribution

All experiments were designed by **Lijie Zuo**. **Shuya Xu** and **Jiaxin Dong** performed the experiments. **Fayin Tang** analyzed the data. Shuya Xu drafted the initial manuscript. **Lihong Li** and **Yunze Guo** conducted the literature search and drafted the manuscript. Shuya Xu and Lijie Zuo edited the manuscript. All authors reviewed the final manuscript.

## Funding

This work was supported by the Natural Science Foundation of Henan (Grant Nos. 252300420667, 262300422210, 262300420486), the Doctoral Start‑up Fund of Henan University of Animal Husbandry and Economy (Grant No. 2022HNUAHEDF009), and the Science and Technology Department of Henan Province (Grant No. 242102310452). Additional financial support was provided by the new round of Key Disciplines (Veterinary Medicine) in Henan Province and the Veterinary Drug Research and Development and Practice Research Teaching Innovation Practice Platform.

## Conflicts of Interest

The authors declare no conflicts of interest.

## Use of Generative AI and AI‐Assisted Technologies in the Writing Process

No generative artificial intelligence or AI‑assisted technologies were used in the design, data analysis, or writing of this manuscript. All content was written and revised by the authors, and the authors take full responsibility for the accuracy and integrity of the work.

## Supporting information




**Supporting File 1**: cbdv71819‐sup‐0001‐SuppMat.docx

## Data Availability

The data that support the findings of this study are available from the corresponding author upon reasonable request.
