# Supplementary material for: Untargeted Metabolomics‐Based Analysis of Metabolic Differences in Different Parts of Bidens bipinnata L
Source: Chem Biodivers. 2026 Oct 15;23(10):e71819. doi: 10.1002/cbdv.71819 (PMC13670983; doi:10.1002/cbdv.71819)
Supplement: Supplementary file 1 [file CBDV-23-e71819-s001.docx]

Supplementary Figure S1. Base peak intensity (BPI) chromatograms of Bidens bipinnata L.

stem (A), fruit (B) and leaf (C) extracts from UPLC-Q-TOF-MS analysis in positive ionization mode.

Supplementary Figure S2. PCA score plot encompassing all QC samples

Supplementary Table S1A. Statistical parameters of differential metabolites in the Stem vs Fruit comparison

| **No.** | **Component name** | VIP | P value | FDR | **\|**Log2FC**\|** |
| --- | --- | --- | --- | --- | --- |
| 1 | L-Phenylalanine | 1.4390 | 9.816×10^-4^ | 0.0031 | -1.1949 |
| 2 | Naringenin | 1.4231 | 1.046×10^-3^ | 0.0032 | 1.0485 |
| 3 | β-Sitosterol | 1.4220 | 1.069×10^-3^ | 0.0033 | 1.2907 |
| 4 | Quercetagetin | 1.4193 | 1.116×10^-3^ | 0.0034 | 1.0529 |
| 5 | Astragalin | 1.4186 | 2.386×10^-3^ | 0.0062 | 1.8308 |
| 6 | Luteolin | 1.4168 | 2.807×10^-4^ | 0.0012 | 2.1563 |
| 7 | Ferulic acid | 1.4158 | 1.024×10^-3^ | 0.0032 | 1.4846 |
| 8 | Quercetin 3-O-β-D-glucofuranoside | 1.4136 | 7.516×10^-4^ | 0.0025 | 2.1468 |
| 9 | Hesperidin | 1.4133 | 2.398×10^-3^ | 0.0062 | 1.1847 |
| 10 | Kaempferol | 1.4127 | 3.074×10^-3^ | 0.0075 | 1.4949 |
| 11 | Bidensynoside A1 | 1.4061 | 1.331×10^-3^ | 0.0039 | -1.1922 |
| 12 | Protocatechuic acid | 1.4060 | 8.353×10^-5^ | 0.0005 | -1.2970 |
| 13 | Quercetin-dimethyl ether | 1.4055 | 1.841×10^-3^ | 0.0051 | -2.1437 |
| 14 | L-Arginine | 1.4052 | 3.341×10^-3^ | 0.0080 | -1.4847 |
| 15 | Kaempferol-3-O-rutinoside | 1.4052 | 7.365×10^-4^ | 0.0025 | 1.4892 |

Supplementary Table S1B. Statistical parameters of differential metabolites in the Stem vs Leaf comparison

| **No.** | **Component name** | VIP | P value | FDR | **\|**Log2FC**\|** |
| --- | --- | --- | --- | --- | --- |
| 1 | Quercetin-3-O-pentose | 1.7061 | 1.711×10^-3^ | 0.0048 | -1.7872 |
| 2 | Naringenin | 1.7031 | 9.890×10^-4^ | 0.0031 | 1.0356 |
| 3 | L-Tyrosine | 1.6967 | 1.042×10^-3^ | 0.0032 | 1.4616 |
| 4 | 1,2-Dihydroxytrideca-3,5,7,9,11-pentayne | 1.6941 | 2.440×10^-3^ | 0.0063 | 1.0391 |
| 5 | L-Phenylalanine | 1.6938 | 7.080×10^-4^ | 0.0024 | -1.6749 |
| 6 | (6E,12E)-tetradecadiene-8,10-diyne-1,3,14-triol | 1.6935 | 1.024×10^-3^ | 0.0032 | 1.4733 |
| 7 | L-Arginine | 1.6927 | 4.578×10^-4^ | 0.0017 | -1.6575 |
| 8 | 7-Phenyl-2-heptene-4,6-diyne-1-ol | 1.6914 | 2.973×10^-4^ | 0.0012 | 1.0297 |
| 9 | Chalcone-α,3,2',4'-hydroxy-2'-O-β-D-glucopyranoside | 1.6895 | 1.317×10^-3^ | 0.0039 | -1.7823 |
| 10 | Kaempferol-3-O-rutinoside | 1.6886 | 1.414×10^-3^ | 0.0041 | 1.6745 |
| 11 | Astragalin | 1.6881 | 2.913×10^-4^ | 0.0012 | -2.7573 |
| 12 | 7-Phenyl-hepta-4,6-diyn-1,2-diol | 1.6877 | 2.786×10^-4^ | 0.0012 | 1.0364 |
| 13 | 7-Hydroxycoumarin | 1.6872 | 2.450×10^-3^ | 0.0063 | 2.697 |
| 14 | Luteolin | 1.6848 | 3.209×10^-3^ | 0.0077 | -1.9188 |
| 15 | Centaurein | 1.6842 | 1.331×10^-3^ | 0.0039 | -1.0348 |

Supplementary Table S1C. Statistical parameters of differential metabolites in the Leaf vs Fruit comparison

| **No.** | **Component name** | VIP | P value | FDR | **\|**Log2FC**\|** |
| --- | --- | --- | --- | --- | --- |
| 1 | Vanillic acid | 2.2190 | 1.338×10^-3^ | 0.0173 | 1.0356 |
| 2 | Phenylheptatriyne (1-phenylhepta-1,3,5-triyne) | 2.2076 | 3.259×10^-4^ | 0.0062 | 1.7883 |
| 3 | Isoleucine | 2.1987 | 1.407×10^-4^ | 0.0035 | -1.0543 |
| 4 | Deca-4,6,8-triyne polyacetylene diglycoside | 2.1951 | 1.477×10^-3^ | 0.0186 | 1.1959 |
| 5 | L-Tryptophan | 2.1879 | 2.385×10^-3^ | 0.0260 | -1.2969 |
| 6 | Kaempferol | 2.1876 | 3.670×10^-4^ | 0.0068 | 1.1849 |
| 7 | Luteoloside | 2.1865 | 1.395×10^-4^ | 0.0035 | 1.8392 |
| 8 | 6-Phenylhexa-1,3,5-triyn-1-yl acetate | 2.1854 | 1.536×10^-4^ | 0.0037 | -1.7829 |
| 9 | tri-O-caffeoylquinic acid | 2.1845 | 1.338×10^-3^ | 0.0173 | 1.4733 |
| 10 | L-Proline | 2.1831 | 2.305×10^-3^ | 0.0255 | -1.6746 |
| 11 | L-Phenylalanine | 2.1776 | 1.489×10^-3^ | 0.0187 | -1.0616 |
| 12 | L-Tyrosine | 2.1771 | 3.279×10^-4^ | 0.0063 | 1.2728 |
| 13 | 2-β-D-Glucopyranosyloxy-1-hydroxy-trideca-3,5,7,9,11-pentayne | 2.1736 | 2.313×10^-3^ | 0.0255 | -1.4637 |
| 14 | Protocatechuic acid | 2.1723 | 3.924×10^-3^ | 0.0374 | 1.9056 |
| 15 | 2-β-D-Glucopyranosyloxy-1-hydroxyltrideca-3,5,7,9,11-pentryne | 2.1711 | 1.440×10^-3^ | 0.0184 | -1.0369 |
